# Effectiveness and cost-effectiveness of meaning-centered group psychotherapy in cancer survivors: protocol of a randomized controlled trial

**DOI:** 10.1002/pon.4726

**Published:** 2018-04-27

**Authors:** Nadia van der Spek, Joël Vos, Cornelia F van Uden-Kraan, William Breitbart, Pim Cuijpers, Kitty Knipscheer-Kuipers, Vincent Willemsen, Rob AEM Tollenaar, Christi J van Asperen, Irma M Verdonck-de Leeuw

**Affiliations:** 1 Department of Clinical Psychology, VU University, VdBoechorststraat 1, room 2B-64, Amsterdam 1081 BT, The Netherlands; 2 Department of Psychology, University of Roehampton, London, UK; 3 Department of Clinical Genetics, Leiden University Medical Center, Leiden, The Netherlands; 4 Department of Otolaryngology – Head & Neck Surgery, VU University Medical Center, Amsterdam, The Netherlands; 5 Department of Psychiatry and Behavioral Sciences, Memorial Sloan-Kettering Cancer Center, New York, USA; 6 Ingeborg Douwes Centrum, Center for psychological care for cancer patients, Amsterdam, The Netherlands; 7 Department of Surgery, Leiden University Medical Center, Leiden, The Netherlands

**Keywords:** Cancer, Survivorship, Meaning, Psycho-oncology, Existential distress, Group psychotherapy, Effectiveness, Cost-effectiveness

## Abstract

**Background:**

Meaning-focused coping may be at the core of adequate adjustment to life after cancer. Cancer survivors who experience their life as meaningful are better adjusted, have better quality of life and psychological functioning. Meaning-Centered Group Psychotherapy for Cancer Survivors (MCGP-CS) was designed to help patients to sustain or enhance a sense of meaning and purpose in their lives. The aim of the proposed study is to evaluate the effectiveness and cost-effectiveness of MCGP-CS.

**Methods/Design:**

Survivors diagnosed with cancer in the last 5 years and treated with curative intent, are recruited via several hospitals in the Netherlands. After screening, 168 survivors are randomly assigned to one of the three study arms: 1. Meaning-Centered Group Psychotherapy (MCGP-CS) 2. Supportive group psychotherapy (SGP) 3. Care as usual (CAU). Baseline assessment takes place before randomisation, with follow up assessments post-intervention and at 3, 6 and 12 months follow-up. Primary outcome is meaning making (PMP, PTGI, SPWB). Secondary outcome measures address quality of life (EORTC-30), anxiety and depression (HADS), hopelessness (BHS), optimism (LOT-R), adjustment to cancer (MAC), and costs (TIC-P, EQ-5D, PRODISQ).

**Discussion:**

Meaning-focused coping is key to adjustment to life after cancer, however, there is a lack of evidence based psychological interventions in this area. Many cancer survivors experience feelings of loneliness and alienation, and have a need for peer support, therefore a group method in particular, can be beneficial for sustaining or enhancing a sense of meaning. If this MCGP-CS is effective for cancer survivors, it can be implemented in the practice of psycho-oncology care.

**Trial registration:**

Netherlands Trial Register, NTR3571

## Background

In the past decade, life expectancies of cancer patients have increased significantly. Due to recent innovations in early detection and treatment, many patients have become cancer survivors and the population of cancer survivors is growing [1,2].

Many cancer patients seem to experience the diagnosis of cancer as a challenge to experiencing life as meaningful, for instance due to shifted priorities in life, or physical hindrances in achieving goals. For some people, the diagnosis of cancer can lead to the experience of life with little or no meaning [3]. Meaning in life is strongly associated to psychological well-being and is liable to alteration after a negative experience like cancer [4–6]. Meaning-focused coping may be at the core of adequate adjustment to cancer: cancer patients who experience their life as meaningful are better adjusted, have better quality of life and psychological functioning [4,7]. Therefore, a meaning-focused psychological intervention might be beneficial for cancer survivors to increase adequate adjustment to life after cancer and prevent and decrease psychological distress.

Several interventions for cancer patients focusing at least partly on experiencing meaning in life have been developed and evaluated. The outcomes of several evaluation studies are promising with improved self esteem, optimism, mood, sense of meaning, spiritual well-being and decreased suffering after intervention. These studies are, however, hampered by methodological limitations, like high dropout rates, no control for the effects of attention, insufficient information on the treatment protocol and short periods of follow up [8–17]. Most of the studied interventions target cancer patients in the palliative phase. None of the described studies assess the cost-effectiveness. To our knowledge there are no randomized controlled trials on meaning-centered psychological interventions targeting cancer survivors.

In the proposed study, we aim to evaluate the effectiveness of a newly developed meaning-centered group psychotherapy for cancer survivors, based on the Meaning-Centered Group Psychotherapy (MCGP) [18]. MCGP, developed by Breitbart and colleagues, is grounded in Frankl’s work and was designed to help patients with advanced cancer to sustain or enhance a sense of meaning, peace and purpose in their lives, despite the confrontation with death [18]. Frankl stated that the will to meaning is the primary motivation of humans [19–21]. He developed a meaning-centered approach in psychotherapy, called *logotherapy*, that focuses on assisting people to detect their individual meaning or purpose in life. A pilot randomized controlled trial showed that MCGP is potentially beneficial for advanced cancer patients for decreasing emotional and spiritual suffering [11].

In the present study, we adapted MCGP for cancer *survivors* (MCGP-CS). Based on outcome of a focus group study on 23 patients [22], and on the input of two psychotherapists with expertise in this specific area, we adjusted the MCGP manual to make it compliant for cancer survivors. Through this focus group study we obtained insight in how survivors experience and talk about meaning in life, and in their perceived need for help with meaning making. In addition, the results indicated that some cancer survivors succeeded in meaning making efforts and experienced sometimes even more meaning in life than before diagnosis, while others struggled with meaning making and expressed an unmet need for help [22]. In preparation of the randomized controlled trial (RCT) studying effectiveness, the feasibility of the MCGP-CS protocol was tested in a feasibility study among 11 participants, divided over two groups. The outcomes of the feasibility study were positive: patient satisfaction and compliance were high [23].

Based on the results of these studies, the MCGP-CS manual and protocol were finalized. An example of an adjustment to Breitbart’s original MCGP for palliative patients addresses attitudinal sources of meaning. In the advanced cancer patient protocol, patients are asked to respond to questions like ‘What would you consider a good or meaningful death?’ ‘How can you imagine being remembered by your loved ones?’ In the adjusted protocol for cancer survivors, they are asked to respond to questions like ‘What are limitations in your life at the moment?’ ‘How can you carry on in life, despite these limitations?’ ‘What do you want to do *now*, that will make you happy and satisfied when you to die later?’ Another change that has been made, based on expert advices, is that every experiential exercise starts with a brief meditation exercise, so feelings can be processed at a deeper level.

The main goal of the present study is to assess effectiveness and cost-effectiveness of MCGP-CS, compared to supportive group psychotherapy (SGP) and to care as usual (CAU) among cancer survivors with psychological or existential distress after treatment and a need for help.

## Methods/Design

### Design

**Figure 1:**
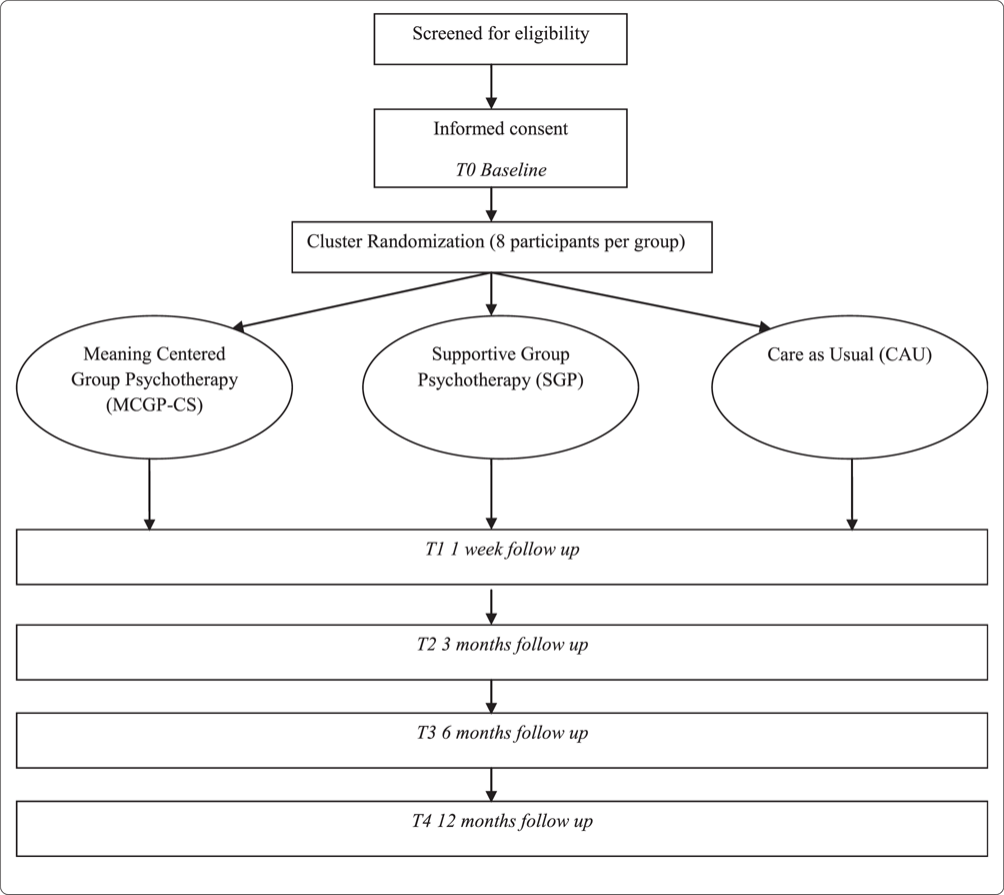
Design of the RCT.

This study is a prospective randomized controlled trial with three study arms: MCGP-CS, SGP and CAU. Cancer survivors are recruited in two different ways: via several hospitals in the Netherlands (region Leiden and Amsterdam) and via public media (i.e. advertisement on websites of patient societies, and in magazines and local newspapers). All cancer survivors who meet in- and exclusion criteria are asked to participate. Survivors are assigned through cluster randomisation to one of the three study arms. The baseline assessment takes place before randomisation, with follow up assessments one week post-intervention and at 3, 6 and 12 months follow-up. Reasons for dropout are registered. The study protocol, information brochure, questionnaires and informed consent form are approved as a multi-center study by the Medical Ethics Committee of the Leiden University Medical Center. The design is illustrated in Figure 1.

### Study sample

#### Inclusion criteria

Cancer diagnosis in the last 5 years, treated with curative intent, main treatment is completed (i.e. surgery, radiotherapy, chemotherapy), ability to attend all therapy sessions, expressed need for psychological help/support and at least one psychosocial complaint (e.g. depressed mood, anxiety, coping issues, life questions, meaning making problems, relationship problems).

#### Exclusion criteria

Severe cognitive impairment, current psychological treatment and insufficient mastery of Dutch language.

The criteria are ascertained during a telephonic interview by a trained psychologist.

A study specific questionnaire comprises questions about sociodemographic (age, gender, religious background, marital status, family situation, education level, other important life events in the past 2 years) and clinical characteristics (type of cancer, cancer treatment, time since treatment) and will be filled out by participants at the first assessment, at baseline. Participants are asked which study-condition has their preference; this will not influence the assignment to the conditions.

### Randomization

Cancer survivors who meet the inclusion criteria and sign the informed consent, are allocated to a group. When the group counts 8 survivors, the group is randomly assigned by an independent researcher, through blocked randomization with randomly selected block sizes, to one of the three study arms.

### Meaning-centered group psychotherapy targeting cancer survivors (MCGP-CS)

Cancer survivors in the experimental study arm participate in MCGP-CS. The main purpose of the MCGP-CS is to sustain or enhance a sense of meaning or purpose in the patient’s life, in order to cope better with the consequences of cancer. MCGP-CS is a manualized 8-week intervention that makes us of didactics, group discussion and experiental exercises that focus around themes related to meaning and cancer survivorship. The sessions take two hours each and are held weekly. The participants use a workbook (called *Life lessons portfolio*) and get homework assignments every week. MCGP-CS is led by one psychotherapist with experience in treating patients with cancer. Each session addresses a specific theme that is related to the concepts and sources of meaning. The themes of the sessions are: 1. Concept and sources of meaning, 2. Meaning before and after cancer, 3. The story of our life as a source of meaning: what made us who we are today, 4. The story of our life as a source of meaning: things we have done and want to do in the future, 5. Attitudinal sources of meaning: encountering life’s limitations, 6. Creative sources of meaning: responsibility, courage and creativity, 7. Experiental sources of meaning, 8. Termination: presentations of our life lessons and goodbyes. Table 1 gives an overview of the themes of each session.

### Supportive group psychotherapy (SGP)

The control condition is an 8-week social support group following Payne et al. [24]. The sessions take two hours and are held weekly. Each group is supervised by a psychotherapist with experience in treating patients with cancer. The psychotherapist has an unconditionally positive regard and empathetic understanding, stimulates patients to actively share their experiences, and focuses on positive emotions, and expression of feelings.

Each of the 8 sessions has a different theme, which is mentioned at the beginning of the session. The themes of the sessions are: 1. group members’ introductions, 2. need for support, 3. coping with medical tests and communicating with physicians, 4. coping with family and friends, 5. coping with work issues, 6. coping with body image and physical functioning, 7. coping with the future, 8. termination: where do we go from here? Table 1 gives an overview of the themes of each session.

### Care as usual (CAU)

Cancer survivors assigned to the CAU study arm do not participate in one of the group interventions. If a patient in the CAU study arm asks the researcher for psychological help, he or she is referred to their General Practitioner (GP). Health care uptake is closely monitored, to enable detailed post-hoc description of what CAU entailed exactly.

### Treatment quality

In the MCGP-CS and the SGP study arms, after each session, the psychotherapist writes a short summary of the session where he/she notes whether the protocol was followed. All group sessions are audio taped and randomly selected audio fragments will be analysed by the researchers to establish whether the therapy protocol was followed correctly.

### Outcome assessment

Outcomes measures include questionnaires on meaning, quality of life, anxiety and depression, hopelessness, optimism, mental adjustment to cancer, satisfaction with the intervention, and sociodemographic and clinical characteristics. Furthermore, a cost-evaluation will be carried out. Patients can choose to complete questionnaires online or via pen and paper. Primary outcomes are collected at all time points (baseline, after one week, 3, 6, and 12 months). Secondary outcome measures are collected at baseline, after one week, 3 and 6 months. Cost evaluation outcomes are collected at baseline, after 3, 6, and 12 months). A complete overview of the outcome measures is presented in Table 2.

### Primary outcome measures

#### Meaning

The Dutch Personal Meaning Profile (PMP) is a 39-item self assessment scale for measuring meaning in life and comprises 5 subscales: religion, dedication to life, fairness of life, goal-orientedness, relationships [25].

The Dutch Post Traumatic Growth Inventory (PTGI) is a 21 item self assessment scale for measuring posttraumatic growth and comprises 5 scales: relationships, viewing new possibilities, personal strength, spirituality, appreciation of life [26].

The Ryff’s Scale of Psychological Well-being (SPWB) is a 49 item questionnaire to assess a person’s level of positive functioning and well-being and comprises 6 scales: autonomy, environmental mastery, personal growth, positive relationships, purpose in life, self-acceptance [27].

### Secondary outcome measures

#### Quality of life

The 30-item EORTC QLQ-C30 (version 3.0) includes a global HRQOL scale (2 items) and comprises 5 functional scales: physical functioning (5 items), role functioning (2 items), emotional functioning (4 items), cognitive functioning (2 items) and social functioning (2 items). There are three symptom scales (nausea and vomiting (2 items), fatigue (3 items) and pain (2 items) and 6 single items relating to dyspnoea, insomnia, loss of appetite, constipation, diarrhoea and financial difficulties [28,29].

### Anxiety and depression

A validated Dutch version of the Hospital Anxiety and Depression Scale (HADS) is used to assess emotional distress. The HADS is a 14-item self-assessment scale for measuring distress with two subscales, anxiety and depression. The HADS was specifically designed for use in the medically ill. The total HADS score ranges from 0 to 42, the subscales from 0 to 21. A score of >15 is used as an indicator of a high level of psychological distress [30].

### Hopelessness

The Dutch Beck Hopelessness Scale (BHS) is a 20 item self-assessment scale for measuring hopelessness. The scale consists of 20 statements about oneself which are endorsed as true or false. The content of 11 statements is hopeless, the content of 9 statements is hopeful [31,32].

### Optimism

The Dutch Life Orientation Test (LOT-R), is a 10-item self-assessment scale for measuring optimism. The scale consists of 10 statements about oneself which are endorsed on a 5-point likert scale (from 1 totally disagree to 5 totally agree) [33,34].

### Adjustment to cancer

Cognitive and behavioural response to cancer diagnosis and treatment is determined by the Mental Adjustment to Cancer (MAC) questionnaire. The MAC scale comprises five subscales: Fighting Spirit, Helplessness/Hopelessness, Anxious Preoccupation, Fatalism and Avoidance [20].

### Satisfaction with the intervention

At T1, cancer survivors in both the MCGP-CS and SGP study arms are asked to evaluate the strengths and weaknesses of the group training that they received and rate their satisfaction with the content, duration, and quality of the training and the trainers on a 15 item likert-scaled questionnaire and on free text responses. Participants in the MCGP-CS condition are asked three additional evaluation questions about the specific content of the MCGP-CS protocol (their opinion on talking about meaning, the homework assignments and the workbook).

### Cost-evaluation

Direct medical and direct non-medical cost data are collected with the Trimbos and iMTA questionnaire on Costs associated with Psychiatric illness (TiC-P) [35,36]. Unit resource use (GP visits, hospital days, etc.) will be multiplied by their appropriate integral cost prices.

An economic evaluation regarding work (loss) and health care use will be conducted as a cost-utility analysis for (changes in) health-related quality of life as assessed with the EQ-5D [37].

Indirect non-medical cost data related to production losses through work loss days and work cutback days will be sampled with the appropriate PRODISQ modules [38]. Indicators of return to work (RTW) are: Time to partial and to full RTW, meaning number of calendar days between end of treatment and first day at work; Time to full RTW corrected for partial RTW.

### Sample size

Based on a priori power analyses for hierarchical multiple regression, assuming a power of .80, Cohen’s d of .80 and alpha of .05, each study condition will need at least 43 cancer survivors. We will anticipate for loss to follow-up of 30%, and will therefore need 56 cancer survivors per condition at baseline. In total, we will recruit 168 cancer survivors during an inclusion period of 2.5 years.

### Statistical analyses

Descriptive statistics, t-tests and Chi^2^ tests will be used to determine whether patient characteristics are similar across experimental conditions. Results will be reported on an intention-to-treat basis. The Linear Mixed Modeling (LMM) procedure will be used to estimate missing values. This procedure includes incomplete cases in the analysis and employs restricted maximum likelihood estimation to calculate parameter estimates. LMM assumes that missing data are missing at random. LMM will be used to investigate the longitudinal development of meaning making in the three groups. The effect of study condition will be tested using contrasts within the LMM. Mediation analyses [39,40] will be used to test as whether development in the patients’ meaning making explains/mediates the expected improvement in psychological functioning in the MCGP condition.

### Economic outcomes

For the economic evaluation we will make use of the pertinent guidelines [36,41–43]. The societal perspective will be taken encompassing intervention costs, direct non-medical costs and indirect costs. The latter is not expected to be very important in the studied population, which is characterised by unemployment, but the data on production losses will be collected anyway. Production losses will be economically valuated using the friction cost method [44]. Costs and effects will be analysed simultaneously, incremental cost-effectiveness ratios (ICERs) will be calculated and placed within 95% confidence intervals, 2,500 bootstrap replications of the ICERs will be projected on a cost-effectiveness plane, ICER acceptability curves will be plotted against different willingness-to-pay ceilings [45], and sensitivity analysis will be conducted as a matter of course focussing on uncertainty in the main cost-drivers. This will be done for the costs per QALY gained in a cost utility analysis.

### Ethical considerations

This study is conducted in accordance with local laws and regulations. Eligible patients are fully informed about the study and asked to participate. The patients receive a patient information sheet and flyer and they are also informed by telephone about the implications of participation. Patients have ample opportunity to ask questions and to consider the implications of the study before deciding to participate. Patients provide written informed consent, compliant with the local and ethical regulations, before participation. Patients are allowed to withdraw from the study without giving a reason, at any time. The study protocol has been approved by the Medical Ethical Committee of Leiden University Medical Center, Leiden, The Netherlands.

## Discussion

The proposed study will assess the effectiveness of MCGP-CS, compared to SGP and to CAU in cancer survivors with psychological or existential distress after treatment. In addition, the cost-effectiveness of MCGP-CS will be determined.

There is a growing need for psychological interventions that target the issues that cancer survivors are dealing with in the aftermath of their disease. Meaning-focused coping is key to adjustment to life after cancer [7,46]. Many cancer survivors experience feelings of loneliness and alienation, and have a need for peer support, therefore a group method in particular, can be beneficial [47]. Group interventions may provide opportunities to cope with these problems. People who benefit from group interventions feel more comforted, less alone and have learned different ways to cope with their situation [47].

To our knowledge there are no RCT’s that evaluate the effectiveness of meaning-centered psychotherapy for cancer survivors. Also, there is little known about who benefits from these types of interventions. Also, there is little known on who benefits from these types of interventions. We want to conduct an RCT that compares MCGP-CS with a SGP that focuses on other issues that cancer survivors deal with (see Table 2). This way, we hope to establish whether a meaning-centered approach is more effective compared to care as usual, than a non-meaning-centered approach. Secondary analyses will be conducted to assess the predictors of effectiveness on an individual level, in order to gain more knowledge on which people benefit the most from the meaning-centered intervention.

To our knowledge, there are no cost evaluations of meaning-centered interventions. Since the number of cancer survivors is increasing rapidly, cost efficient psychological care is, from an economic point of view, important to warrant the feasibility of implementation in mental health care settings.

This study evaluates if MCGP-CS is effective for cancer survivors and if so, whether this is a cost efficient method. If this MCGP-CS is effective for cancer survivors, it can be implemented in the practice of psycho-oncology care. The broad collaboration in this project with several hospitals and psycho-oncology organisations, facilitates possible implementation in practice after this evaluation. There are few evidence based group intervention manuals available for cancer patients. For meaning-centered group psychotherapy for cancer survivors, there are no evidence based intervention manuals yet. Therefore, if the results of this RCT are positive on effectiveness measures, the intervention protocol can be an important addition to the advancement of evidence based psychological care for cancer patients.

## Figures and Tables

**Table 1:** Session topics covered in MCGP-CS^1^ and SGP^2^

Session	MCGP-CS	SGP
1	Concept and sources of meaning	Group member introductions
2	Meaning before and after cancer	The need for support
3	The story of our life as a source of meaning: what made us who we are today	Coping with the medical test and communicating with providers
4	The story of our life as a source of meaning: things we have done and want to do in the future	Coping with family and friends
5	Attitudinal sources of meaning: encountering life’s limitations	Coping with vocational issues
6	Creative sources of meaning: responsibility, courage and creativity	Coping with body image and physical functioning
7	Experiental sources of meaning	Coping with the future
8	Termination: presentations of our life lessons and goodbyes	Termination: Goodbyes and how do we go from here?

1 Meaning-Centered Group Psychotherapy for Cancer Survivors.

2 Supportive Group Psychotherapy.

**Table 2:** Outcome measures and instruments

Outcome measures	Instrument
*Primary*^1^	
Meaning	Personal Meaning Profile (PMP) [24]
Post Traumatic Growth	Post Traumatic Growth Inventory (PTGI) [25]
Positive psychological functioning and wellbeing	Ryff’s Scale of Psychological Well-being (SPWB) [26]
*Secondary*^2^	
Quality of life	30-item EORTC QLQ-C30 (version 3.0) [27,28]
Anxiety and Depression	Hospital Anxiety and Depression Scale (HADS) [29]
Hopelessness	Beck Hopelessness Scale (BHS) [30,31]
Optimism	Life Orientation Test (LOT-R) [31,32]
Adjustment to cancer	Mental Adjustment to Cancer (MAC) [20]
*Cost evaluation*^3^	Trimbos and iMTA questionnaire on Costs associated with Psychiatric illness (TiC-P) [34,35]
	EQ-5D [36]
	PRODISQ [37]

1 Assessment at T0, T1, T2, T3, T4.

2 Assessment at T0, T1, T2, and T3.

3 Assessment at T0, T2, T3 and T4.
